# Are diagnoses of unruptured intracranial aneurysms associated with quality of life, psychological distress, health anxiety, or use of healthcare services in untreated individuals? A longitudinal, nested case-control study

**DOI:** 10.1016/j.bas.2024.102915

**Published:** 2024-08-13

**Authors:** Ingvild M. Rosenlund, Tor Ingebrigtsen, Liv-Hege Johnsen, Unni Ringberg, Ellisiv B. Mathiesen, Jørgen Isaksen

**Affiliations:** a University Hospital of North Norway, Department of Neurosurgery, Ophthalmology, and Otorhinolaryngology, Tromsø, Norway; b UiT the Arctic University of Norway, Faculty of Health Sciences, Department of Clinical Medicine, Tromsø, Norway; c University Hospital of North Norway, Department of Radiology, Tromsø, Norway; d UiT the Arctic University of Norway Faculty of Health Sciences, Medical Education Unit, Tromsø, Norway; e University Hospital of North Norway, Department of Neurology, Tromsø, Norway

**Keywords:** Screening, Incidental findings, Intracranial aneurysm, Quality of life, Psychological stress, Services utilisation

## Abstract

**Introduction:**

Increasing imaging examination rates leads to a corresponding rise in the detection rates of unruptured intracranial aneurysms (UIAs). There is limited knowledge on how the detection of UIA affects health-related outcomes in untreated patients.

**Research question:**

Is the diagnosis of UIA associated with psychosocial outcomes, healthcare services utilisation, or sick leave in untreated individuals?

**Material and methods:**

Nested case-control study with 96 participants diagnosed with UIAs through magnetic resonance angiography (MRA) screening, not receiving preventive aneurysm obliteration. Comparisons were made with Control1 (192 participants with negative MRAs) and Control2 (192 individuals not MRA screened). Quality of life, psychological distress, and health anxiety were assessed using EQ-5D-5L including EQ VAS, Hopkins Symptom Checklist-10, and Whiteley Index-6, respectively. Healthcare service utilisation and sick leave was measured using registry data. Median follow-up was 32–55 months for the different outcomes.

**Results:**

UIA were in general not associated with psychosocial outcomes, neither compared to pre-screening values nor to controls. The exemption was a lower mean EQ VAS score at follow-up for cases (76.7) versus Control1 (80.0), regression coefficient −3.87 (95% CI (−7.60, −0.14). Cases had significantly higher rates of radiology exams compared to controls, with 1.47 (95% CI 1.25, 1.74) exams per person-year versus 0.91 (C95% CI 0.75, 1.09) for Control1 and 0.95 (95% CI CI 0.79, 1.14) for Control2. No significant differences were observed in other psychosocial outcomes, healthcare services utilisation, or sick-leave.

**Discussion and conclusions:**

The overall impact of untreated UIAs appears to be limited when assessed years after diagnosis.

## Highlights

• This study is the first to assess outcomes before and after the diagnosis of unruptured intracranial aneurysms (UIA).
• UIA detection was associated with significantly higher radiology exams rates compared to control groups.
• UIA detection was not associated with the use of other health care services, long-term psychosocial outcomes, or sick leave.

## 1 Introduction

### 1.1 Background

Unruptured intracranial aneurysms (UIA) are relatively common, with a prevalence of 3–7% in the general population, estimates varying based on size and location definitions (Vlak et al., 2011; Johnsen et al., 2022). UIA may rupture, causing severe subarachnoid haemorrhage (Macdonald and Schweizer, 2017). Preventive treatment options include surgical clipping and endovascular coiling, both of which are associated with serious complication rates exceeding 5% (Algra et al., 2019, Thompson et al., 2015). The rupture risk for UIA depends on size and location, with an overall risk of approximately 1% after 10 years of follow-up (Wermer et al., 2007), notably lower for UIAs <3 mm (Malhotra et al., 2017). Due to the low rupture risk and the associated risks of aneurysm obliteration, many patients are managed conservatively with radiologic follow-up and risk factor modification, instead of invasive treatment (Thompson et al., 2015).

Screening for UIA is not recommended for the general public (Thompson et al., 2015). In practice, opportunistic screening is becoming more prevalent, with the rise of private providers promoting health checks that include magnetic resonance imaging of the head (Morita, 2019, Scan.com. Available from). Concurrently, diagnostic imaging examination rates have consistently risen for decades (Smith-Bindman et al., 2019, World Health Organization), with enhanced detection of UIAs (Gabriel et al., 2010).

There is little knowledge on how the detection of UIA affects health-related quality of life (HRQOL), psychological distress, and health anxiety in untreated patients (Ignacio et al., 2022). Previous studies, limited to one case-control study with 31 participants and a few cross-sectional and cohort studies, have reported varying degrees of negative impact on these factors (Ignacio et al., 2022; Algra et al., 2022). To our knowledge, there are no studies that have assessed psychosocial outcomes in the same individual before and after UIA detection.

How findings of UIA affect use of healthcare services and work absenteeism is understudied. As studies indicate reduced psychosocial wellbeing (Ignacio et al., 2022; Algra et al., 2022), it is plausible to assume that such a diagnosis might influence use of healthcare services.

### 1.2 Study objectives

To determine if screening diagnoses of UIA are associated with.

(1) Health-related quality of life, health anxiety, or psychological distress in untreated patients.
(2) Use of healthcare services or sick-leave in untreated patients.

## 2 Methods

### 2.1 Design

This is a longitudinal, population-based nested case-control study with participants recruited from the Tromsø Study. The STROBE statement guided the conduct and reporting (von Elm et al., 2008).

### 2.2 Setting

The Tromsø Study is a population-based repeated cross-sectional health survey (Jacobsen et al., 2012). Tromsø7 (2015–2016) comprised two visits. The first visit included questionnaires and basic measurements. All inhabitants ≥40 years (n = 32 591) in Tromsø were invited and 65% (n = 21 083) consented. A subset (n = 13 028) of randomly selected participants in different age groups (n = 9125) and participants who had attended the second visit during Tromsø6 (n = 3103) were pre-marked for a next visit. Participation in the first visit was required for invitation to the second visit. Of 9253 invited, 8346 attended.

At the second visit, participants underwent various examinations (Hopstock et al., 2022), including carotid artery ultrasound for a subset (n = 3027). Those who had ultrasound were invited to magnetic resonance angiography (MRA) of the brain. A total of 1877 (62%) participated, and were scanned with a 3 T S Skyra MR. Three-dimensional time-of-flight sequences were used for aneurysm detection, defined as intradural aneurysms ≥1 mm, as previously described (Johnsen et al., 2022). Out of 1862 MR A exams of sufficient quality, 143 (8.5%) were diagnosed with UIAs. Feedback was not given to 42 individuals, based on an assessment that communicating the diagnosis would not significantly benefit them.

The remaining (n = 101) attended the neurosurgery outpatient clinic at the University Hospital of North Norway. After individual assessment, including PHASES scores (Greving et al., 2014), preventive aneurysm obliteration was offered to 5 individuals. The other 96 received diagnosis information, advice on smoking cessation and blood pressure control, and a follow-up regimen, including imaging.

Norway's public healthcare system ensures universal access. Private health insurance covered only 10% of the population in 2017 (Grepperud, 2018). Hospital admissions are free of charge, while primary and specialized outpatient healthcare require a small co-payment (Helfo, 2023).

## 3 Participants

The study population is Tromsø7 participants who were diagnosed with and informed about UIAs without receiving preventive aneurysm obliteration (n = 96). There are two control groups; Control1 are MRA-screened participants without a UIA diagnosis. Control2 represent the non-screened population, including Tromsø7 participants who attended the second visit, but were not invited to MRA. Both control groups are matched 2:1 with cases by sex and age. Eligible participants were contacted by mail. Written informed consent was obtained.

### 3.1 Outcome variables and data sources

The outcome variables were: HRQOL, psychological distress, health anxiety, medication use, general practitioner (GP) appointments, laboratory services, radiology exams, appointments at physiotherapists or chiropractors, reimbursements, hospital admissions, outpatient specialist appointments, and sick leave.

### 3.2 Tromsø7 and follow-up questionnaire

To assess baseline comparability, the Tromsø Study provided data on age, sex, baseline measurements of blood pressure, body mass index (BMI), cholesterol, and HbA1c and self-reported medical history, smoking status, education level, and household income.

In Tromsø7, HRQOL, psychological distress, and health anxiety were measured by EQ-5D-5L (Rabin and de Charro, 2001), Hopkins Symptom Checklist-10 (HSCL-10) (Strand et al., 2003), and the Whiteley Index-6 (WI-6) (Veddegjaerde et al., 2014), respectively. Participants also answered questions on medication usage.

The EQ-5D-5L has five dimensions on a 5-point Likert scale, yielding EQ-index scores ranging from −0.285 to 1, where 1 signifies perfect health (Devlin et al., 2018). Additionally, EQ VAS rates self-rated health from 0 to 100.

HSCL-10 consists of ten questions on a 4-point Likert scale, with sum scores ranging from 1 to 4. Mean scores ≥1.85 are indicative of psychological distress (Strand et al., 2003). WI-6 has six questions on a 5-point Likert scale, yielding sum scores from 0 (none) to 24 (most severe level of health anxiety).

We used baseline data from Tromsø7, and the participants completed the same questionnaires for follow-up. Study entry was defined as the date of the MR result letter, from January 2016 to July 2020. Follow-up ended when the questionnaires were completed, between April and August 2021. The median follow-up was 48 months (range 10–63).

### 3.3 Norway Control and Payment of Health Reimbursement Database

We obtained data from the Norway Control and Payment of Health Reimbursement Database (KUHR) on publicly funded healthcare provided by general practitioners (GPs), physiotherapists, chiropractors, outpatient radiology centres, outpatient laboratory services, reimbursements, and deductibles. KUHR provided data from study entry until December 31, 2020, with a median follow-up of 43 months (range 6–59).

We defined GP appointments as any patient contact recorded in a reimbursement claim from a GP. This includes regular in-person, online, and telephone consultations, and isolated procedures at the GP's office. We analysed the use of physiotherapists and chiropractors together, defined by a reimbursement bill from either.

Laboratory services in primary care was defined by a code for laboratory work (701a) in GP bills, according to the Tariff for general practitioners (The Norwegian Medical Association, 2022). Outpatient radiology exams were defined by reimbursement claims from outpatient radiology departments, using Norwegian Classification of Radiological Procedures codes (The Norwegian Directorate of eHealth, 2018). We analysed the number of claims with code 701a or radiological procedure codes, as the number of individual services per bill is not available.

We analysed total reimbursements from KUHR and deductibles, regardless of the care provider.

### 3.4 The Norwegian Patient Registry

The Norwegian Patient Registry (NPR) provided data on all hospital admissions and outpatient appointments within publicly funded specialized healthcare. Hospital admissions and outpatient appointments were defined as discharges with length of stay >8 and ≤8 h, respectively. NPR provided data from study entry until December 31, 2021, with a median follow-up of 55 months (range 10–71).

### 3.5 Statistics Norway

Data on doctor-certified sick leave were obtained from Statistics Norway (SSB). Participants above retirement age (≥67 years) were excluded, resulting in a sample of 210 participants (42 cases and 168 controls) who were followed until they turned 67. Data covered the period from study entry until December 31st, 2019, with a median follow-up of 32 months (range 9–47).

### 3.6 Statistical analyses

We conducted separate analyses comparing a): cases to Control1 and b): cases to Control2. For baseline characteristics, we used chi-square for binary variables and two-sample *t*-test for continuous variables.

Differences in EQ-Index and EQ VAS between groups at follow-up were tested with linear regression. As there was no available value set specific to Norway, we utilised the English version to calculate the EQ-Index (Devlin et al., 2018). We used logistic regression to test for differences between groups in the proportion reporting any problem for each dimension of EQ-5D-5L.

For both HSCL-10 and WI-6, we calculated sum scores and used linear regression to test for differences between groups. For HSCL-10, we also obtained the proportions who had sum scores above 1.85 and used logistic regression to test for group differences.

We quantified self-reported current use of blood pressure-lowering drugs, cholesterol-lowering drugs, diuretics, drugs for heart disease, diabetes medications, drugs for hypothyroidism, painkillers, sleeping pills, tranquilizers, and antidepressants. We used logistic regression to test for group differences at follow-up. All regression analyses for EQ-5D-5L, HSCL-10, WI-6, and medication usage were adjusted for corresponding baseline values (e.g., baseline EQ-Index for EQ-Index analysis).

For KUHR, NPR, and SSB variables we quantified the use of healthcare services, reimbursements, and sick leave per person-year during the follow-up period. We used Poisson regression models with robust variance estimator for continuous variables and chi-square tests to compare the proportions utilising various services.

Missing values in the questionnaire data ranged from 0 to 9% per item. Many missing values seemed to be due to participants only marking the medications and psychosocial issues they had, leaving other fields blank (not marking 'no use/problem'), and were thus not missing at random. However, the proportion of missing values did not differ significantly between groups; hence, missing values were excluded from statistical analyses. Sensitivity analyses, where missing outcome values were counted as 'no use/problem', did not significantly alter the group comparisons. All analyses applied a 95% confidence interval (CI). We used StataMP 17 for statistical analyses (StataCorp., 2019).

## 4 Results

### 4.1 Baseline characteristics

Among 480 participants, 59% were women (Table 1). Mean age at study entry was 65 years. Mean systolic blood pressure at baseline was 135.9 mmHg for cases, 130.8 mmHg for Control1 and 133.6 mmHg for Control2, differences were not statistically significant. Baseline values for diastolic blood pressure, BMI, laboratory results, and medical history showed minimal differences across groups. A higher percentage of cases (56.4%) compared to Control1 (42.4%) were previous smokers (p = 0.026). Current smoking, alcohol consumption, household income, and education level were consistent across groups.

**Table 1:** Baseline characteristics.

	Cases	Control1	P-value^a^	Control2	P-value^a^
Participants, n	96	192		192	
Women, n (%)	57/39 (59)	114 (59)		114 (59)	
Age at study entry, year mean/median (range)	65.2/66.5 (42–86)	65.2/66.5 (42–86)		65.2/66.5 (42–86)	
**Aneurysm characteristics**					
Aneurysm size, median/mean mm (SD, range)	3.1/3.6 (1.8, 1–11.6)	N/A		N/A	
Saccular aneurysm, n (%)	91 (94.8)	N/A		N/A	
Fusiform aneurysm, n (%)	5 (5.2)	N/A		N/A	
**Baseline measurements**					
Systolic blood pressure, mean mmHg (SD)	135.9 (21.0)	130.8 (22.4)	0.066	133.6 (19.7)	0.36
Diastolic blood pressure, mean mmHg (SD)	73.8 (9.5)	73.5 (9.4)	0.79	75.2 (10.1)	0.26
BMI, mean (SD)	26.8 (4.7)	26.5 (3.8)	0.55	27.5 (4.7)	0.20
Total cholesterol, mean mmol/l (SD)	5.5 (1.1)	5.6 (1.0)	0.55	5.5 (1.2)	0.77
LDL cholesterol, mean mmol/l (SD)	3.5 (1.0)	3.7 (1.0)	0.30	3.6 (1.1)	0.89
HDL cholesterol, mean mmol/l (SD)	1.7 (0.50)	1.6 (0.48)	0.29	1.6 (0.52)	0.34
HbA1c, mean % (SD)	5.8 (0.52)	5.8 (0.56)	0.61	5.8 (0.75)	0.54
**Medical history, present or previous**					
Hypertension, n/total (%)	36/92 (39.1)	58/185 (31.4)	0.20	54/184 (29.4)	0.10
Myocardial infarction, n/total (%)	6/90 (6.7)	9/183 (4.9)	0.55	4/185 (2.2)	0.061
Atrial fibrillation, n/total (%)	7/91 (7.7)	13/182 (7.1)	0.74	12/182 (6.6)	0.74
Angina pectoris, n/total (%)	7/91 (7.7)	6/182 (3.3)	0.11	5/184 (2.7)	0.057
Stroke, n/total (%)	3/90 (3.3)	2/181 (1.1)	0.20	8/185 (4.3)	0.69
Cancer, n/total (%)	12/93 (12.9)	24/184 (13.0)	0.97	20/186 (10.8)	0.60
Daily smoking, present, n/total (%)	18/94 (19.2)	25/191 (13.1)	0.18	29/190 (15.3)	0.41
Daily smoking, previous (not present), n/total (%)	53/94 (56.4)	81/191 (42.4)	0.026	98/190 (51.6)	0.45
*Alcohol frequency*					
Never	3/95 (3.2)	17/191 (8.9)		12/189 (6.3)	
Monthly or less frequently	24/95 (25.3)	50/191 (26.2)		49/189 (25.9)	
2-4 times a month	41/95 (43.2)	58/191 (30.4)		74/189 (39.2)	
2-3 times a week	16/95 (16.8)	50/191 (26.2)		45/189 (23.8)	
4 or more times a week	11/95 (11.6)	16/191 (8.4)		9/189 (4.8)	
Ordinal logistic regression, coefficient (95% CI)		−0.11 (−0.55, 0.33)	0.63	−0.18 (−0.63, 0.27)	0.44
*Alcohol units usually consumed when drinking*					
1–2	65/91 (71.4)	121/174 (69.5)		123/179 (68.7)	
3–4	19/91 (20.9)	42/174 (24.1)		43/179 (24.0)	
5–6	5/91 (5.5)	11/174 (6.3)		10/179 (5.6)	
7–9	2/91 (2.2)	0/174 (0)		2/179 (1.1)	
10 or more	0/91 (0)	0/174 (0)		1/179 (0.6)	
Ordinal logistic regression, coefficient (95% CI)		0.06 (−0.50, 0.61)	0.84	0.11 (−0.44, 0.66)	0.69
**Socioeconomic status**					
*Household income category, NOK*					
<150000, n/total (%)	1/89 (1.1)	1/179 (0.56)		2/177 (1.1)	
150000-2500000, n/total (%)	10/89 (11.2)	10/179 (5.6)		12/177 (6.8)	
251000-350000, n/total (%)	7/89 (7.9)	20/179 (11.2)		26/177 (14.7)	
351000-450000, n/total (%)	11/89 (12.4)	18/179 (10.1)		20/177 (11.3)	
451000-550000, n/total (%)	7/89 (7.9)	25/179 (14.0)		22/177 (12.4)	
551000-750000, n/total (%)	17/89 (19.1)	29/179 (16.2)		31/177 (17.5)	
751000-1000000, n/total (%)	17/89 (19.1)	32/179 (17.9)		38/177 (21.5)	
>1000000, n/total (%)	19/89 (21.4)	44/179 (24.6)		26/177 (14.7)	
Ordinal logistic regression, coefficient (95% CI)		0.15 (−0.30, 0.60)	0.52	−0.18 (−0.64, 0.27)	0.43
*Highest level of completed education*					
Primary/partly secondary education. (Up to 10 years of schooling), n/total (%)	33/95 (34.7)	60/190 (31.6)		64/189 (33.9)	
Upper secondary education. (A minimum of 3 years), n/total (%)	30/95 (31.6)	43/190 (22.6)		54/189 (28.6)	
College/university less than 4 years, n/total (%)	15/95 (15.8)	36/190 (19.0)		34/189 (18.0)	
College/university more than 4 years, n/total (%)	17/95 (17.9)	51/190 (26.8)		37/189 (19.6)	
Ordinal logistic regression, coefficient (95% CI)		0.35 (−0.10, 0.79)	0.13	0.10 (−0.35, 0.54)	0.67

BMI, body mass index; HbA1c, haemoglobin A1C; HDL, high-density lipoprotein; LDL, low-density lipoprotein; NOK, Norwegian kroner; SD, standard deviation.

a Separate analyses of differences between cases and the two control groups; chi-square tests for binary variables, two-sample t-tests for continuous variables, and ordinal logistic regression for ordinal variables.

### 4.2 Health-related quality of life, psychological distress, and health anxiety

At follow-up 15 (3.1%) of 480 participants were unreachable due to incorrect addresses, severe dementia, or death. Of those invited, 79% (367/465) consented and completed the follow-up questionnaire. A higher percentage of cases (76/91, 84%) concented compared to Control1 (152/188, 81%) and Control2 (139/186, 75%). Among respondents, women comprised 55% of cases and 57% of control groups. Mean age at study entry was 65 years.

Respondents in all groups reported consistently high HRQOL, with median EQ-index 0.937 and median EQ-VAS 80–82.5 at both baseline and follow-up (Table 2). In regression analyses on differences between groups at follow-up, adjusted for baseline values, cases had a significantly lower EQ VAS compared to Control1. Cases showed a decrease in mean EQ VAS from 78.5 at baseline to 76.7 at follow-up, while Control1 experienced a slight increase from 77.6 to 80.0 (regression coefficient −3.87 (95% CI -7.60, −0.14)). Control2 also had a slight increase in mean EQ VAS over time, but differences compared to cases were not statistically significant. There were no statistically significant differences between the groups in any of the separate EQ-5D-5L dimensions.

**Table 2:** Health-related quality of life, psychological distress and health anxiety following diagnosis of unruptured intracranial aneurysm.

	Cases		Control1		Regression coefficient (95% CI)^a^	Control2		Regression coefficient (95% CI)^a^
	Baseline	Follow-up	Baseline	Follow-up		Baseline	Follow-up	
*EQ-5D-5L*								
EQ-Index, median (IQR)	0.937 (0.121)	0.937 (0.077)	0.937 (0.117)	0.937 (0.140)	−0.008 (−0.032, 0.016)	0.937 (0.082)	0.937 (0.140)	−0.0004 (−0.029, 0.028)
Missing EQ-Index, n (%)	5 (6.6)	5 (6.6)	4 (2.6)	4 (2.6)		9 (6.5)	9 (6.5)	
EQ VAS, median (IQR)	80.0 (17.5)	80.0 (20)	80.0 (20.0)	80.0 (15.0)	−3.87 (−7.60, −0.14)	80.0 (20.0)	82.5 (20.0)	−3.02 (−7.61, 1.57)
Missing EQ VAS, n(%)	4 (5.3)	4 (5.3)	6 (3.9)	6 (3.9)		11 (7.9)	11 (7.9)	
*Hopkins Symptom Checklist-10*								
Sum score, median (IQR)	1.20 (0.40)	1.20 (0.40)	1.10 (0.40)	1.10 (0.30)	0.061 (−0.019, 0.14)	1.15 (0.40)	1.10 (0.40)	0.061 (−0.020, 0.14)
Above cut-off, n/total (%)	3/66 (4.6)	6/66 (9.1)	8/141 (5.7)	9/141 (6.4)	0.52 (−0.64, 1.68)	11/126 (8.7)	7/126 (5.6)	1.21 (−0.24, 2.66)
Missing, n (%)	10 (13.2)	10 (13.2)	11 (7.2)	11 (7.2)		13 (9.4)	13 (9.4)	
*Whitley Index-6*								
Sum score, median (IQR)	2 (3.0)	3 (4.5)	2 (4.0)	2 (4.0)	0.56 (−0.15, 1.27)	2 (5.0)	3 (6.0)	0.27 (−0.57, 1.12)
Missing, n (%)	4 (5.3)	4 (5.3)	9 (5.9)	9 (5.9)		9 (6.5)	9 (6.5)	

CI, confidence interval; IQR, interquartile range.

a Separate regression analyses of differences between cases and the two control groups at follow-up, adjusted for corresponding baseline values; linear regression for continuous variables and logistic regression for the binary variable (above cut-off). The regression coefficient represents the difference in outcome for the cases compared to the control group.

Across all groups, there were minimal changes over time in mean HSCL-10 score, and there were few with scores indicative of psychological distress. The majority reported minimal health anxiety both at baseline and follow-up. There were no statistically significant differences between groups in either HSCL-10 or WI-6 (Table 2).

### 4.3 Use of medication

The use of medication was similar between the groups with no statistically significant differences (Supplemental file 1).

### 4.4 Use of healthcare services

Significantly higher rates of radiology exams were observed for cases compared to both control groups, with 1.47 (95% CI 1.25, 1.74) exams per person-year for cases, and 0.91 (95% CI 0.75, 1.09) for Control1, and 0.95 (95% CI 0.79, 1.14) for Control2, respectively (Table 3). While 97% (n = 93) of cases underwent a radiology exam, 72% (n = 138) and 70% (n = 134) of Control1 and Control2 were examined, respectively (p < 0.000). There were no notable differences in use of other healthcare services.

**Table 3:** Use of healthcare services following diagnosis of unruptured intracranial aneurysm.

	Cases	Control1		Control2	
			P-value^a^		P-value^a^
Hospital admissions per person-years, mean (95% CI)	0.26 (0.19, 0.36)	0.22 (0.16, 0.30)	0.44	0.33 (0.24, 0.44)	0.33
Outpatient appointments per person-years, mean (95% CI)	3.75 (3.06, 4.61)	3.30 (2.60, 4.19)	0.43	3.30 (2.70, 4.03)	0.38
GP appointments per person-year, mean (95% CI)	10.3 (8.94, 11.8)	9.60 (8.52, 10.8)	0.46	10.1 (8.85, 11.5)	0.85
Physiotherapy or chiropractic appointments per person-year, mean (95% CI)	3.06 (1.81, 5.18)	2.73 (1.72, 4.36)	0.75	3.23 (2.14, 4.86)	0.88
Radiology exams per person-year, mean (95% CI)	1.47 (1.25, 1.74)	0.91 (0.75, 1.09)	0.000	0.95 (0.79, 1.14)	0.001
Laboratory services, bills per person-year, mean (95% CI)	2.73 (2.25, 3.31)	2.60 (2.25, 3.01)	0.70	2.48 (2.12, 2.91)	0.46
Deductibles per person-year, mean NOK (95% CI)	2062 (1852, 2296)	1698 (1544, 1867)	0.008	1747 (1591, 1919)	0.023

CI, confidence interval; NOK, Norwegian kroner.

a Separate Poisson regression analyses of differences between cases and the two control groups.

### 4.5 Sick leave

The median age at study entry was 60 years (range 42–66). Among cases, 29% had a sick leave during the study period, compared to 17% in Control1 and 23% in Control2 (Table 4). Although the cases had higher number of sick leave days per person-year compared to controls, with 18.9 days (95% CI 9.8, 36.1) versus 8.9 days (95% CI 4.4, 17.9) for Control1 and 14.6 days (95% CI 9.1, 23.6) for Control2, these differences were not statistically significant.

**Table 4:** Sick leave following diagnosis of unruptured intracranial aneurysm.

	Case	Control1		Control2	
			P-value^a^		P-value^a^
Number of participants with a sick leave during study period, n (%)	12 (28.6)	14 (16.7)	0.12	19 (22.6)	0.47
Sick leave, days per person-year (95% CI)	18.9 (9.8, 36.1)	8.9 (4.4, 17.9)	0.12	14.6 (9.1, 23.6)	0.54
Sick days reimbursed by the state per person-year (95% CI)	12.5 (6.5, 24.3)	5.3 (2.5, 11.4)	0.095	9.1 (5.4, 15.4)	0.46
Reimbursement by the state for sick leave per person-year, NOK (95% CI)	15516 (7969, 30210)	6057 (2865, 12804)	0.066	9040 (5327, 15343)	0.21

CI, confidence interval; NOK,Norwegian kroner.

a Separate analyses of differences between cases and the two control groups; chi-square tests for binary variables and Poisson regression analyses for continuous variables.

## 5 Discussion

### 5.1 Key results

The awareness of screening diagnoses of UIA in untreated patients was in general not associated with differences in psychosocial outcomes, either compared to pre-screening values or to controls. The exception was a statistically significant lower EQ VAS score when compared to controls with negative MRAs. However, the absolute differences were minimal, and other EQ-5D-5L scores remained consistent.

Screening diagnoses of UIA in untreated patients were associated with significantly higher rates of radiology exams. No other statistically significant differences were found in use of healthcare services or work-related outcomes. Noteworthy, individuals with UIA had more than double the sick leave days per person-year compared to controls, although these differences were not statistically significant.

### 5.2 Limitations

Both cases and controls were chosen from participants who voluntarily took part in numerous screening exams in Tromsø7, which may introduce selection bias and reduce generalisability. Previous studies indicate that health survey participants tend to have higher socioeconomic status and lower mortality compared to non-responders, a trend also observed in earlier Tromsø Studies (Jacobsen et al., 2012; Eggen et al., 2013). These differences in sociodemographic profiles could influence the interpretation of subjective outcomes like HRQOL. Therefore, the findings of our study may not be fully applicable to general populations beyond the health survey context. However, both the Tromsø Study and our study achieved high participation rates, enhancing the likelihood that our findings can be reasonably extrapolated to similar populations with comparable healthcare systems and sociodemographic profiles.

The outcomes we studied could be influenced by other screening exams in Tromsø7. Nevertheless, a previous study found no such associations (submitted, unpublished data), supporting our results.

Performing multiple tests raises the likelihood of chance findings. A slight disparity in EQ VAS was observed between cases and Control1. These minor differences may represent type 1 error, particularly given their limited impact in absolute numbers.

Our sample size is relatively small, introducing the risk of type 2 errors. To enhance statistical power, we employed a 1:2 ratio when matching cases to controls. Nevertheless, actual variances may not attain statistical significance due to the small sample size. This consideration is especially important in the analysis of sick leave, with only 42 cases, and substantial observed differences that did not reach statistical significance.

Due to the relatively small sample size, we were not able to do sex- and age-stratified analyses. The median age at study entry was 66 years. Hence, our findings might not be representative for subgroups, including younger populations.

In the absence of recommended measures tailored for patients with UIAs (Ignacio et al., 2022), we used generic questionnaires to assess psychosocial outcomes. These generic instruments may lack content validity and may have limited sensitivity in detecting psychosocial outcomes related to screening diagnosis (McCaffery and Barratt, 2004). Furthermore, we only used quantitative measures, whereas qualitative research has identified psychological harms and changes in activities of daily living in untreated patients with UIA that quantitative measures have not captured (Jelen et al., 2023).

### 5.3 Interpretation

While our study revealed elevated rates of radiology exams and associated higher deductible rates, the overall impact of the examined outcomes appears limited. We interpret the heightened frequency of radiological examinations directly to the identification of UIAs, prompting recommended surveillance.

In our study, we found a minimal negative impact on HRQOL for patients with untreated UIA, as measured by EQ VAS, while observing no noticeable impact on other psychosocial outcomes. The observed decrease in EQ VAS was well below the minimal clinically important difference of >7 points estimated for other neurological conditions (Chen et al., 2016, Randall et al., 2022). This suggests that the slight reduction in EQ VAS is unlikely to be of clinical relevance.

Our results contrast with previous studies reporting negative impacts on different psychosocial outcomes (Ignacio et al., 2022; Jelen et al., 2023; Buijs et al., 2011; van der Schaaf et al., 2002; Lemos et al., 2020; Su et al., 2014; Li et al., 2017). However, previous studies exhibits significant heterogeneity both in results and methodology (Ignacio et al., 2022). Lemos et al. found anxiety in 27% of persons with UIA, but minimal depression, and no reduction in HRQOL compared to the general population when measured 3 years after diagnosis (Lemos et al., 2020). Conversely, Buijs et al. showed reduced HRQOL but no effect on mood when assessed 4 years after diagnosis (Buijs et al., 2011). Likewise, Li et al. reported reduced HRQOL and no effect on mood. Additionally, they showed a higher HRQOL for patients diagnosed 5 years ago compared to those diagnosed 1 year ago (Li et al., 2017). Su et al. reported anxiety and depression in 84% and 71% of UIA patients, respectively, 1 year after diagnosis, compared to 16% and 44 % in the general population. They also demonstrated an initial decline in HRQOL. However, after 5 years, neither HRQOL, anxiety, nor depression showed differences from the general population (Su et al., 2014).

We used EQ-5D, WI-6, and HCSL-10 for assessment of psychosocial outcomes, whereas other studies have used various other instruments (Ignacio et al., 2022), making direct comparison difficult. Our results, along with those of previous studies, highlight the challenges in using different generic questionnaires not validated specifically for UIA patients when assessing psychosocial implications.

Moreover, the median follow-up time in our study was four years. Our findings regarding minimal psychosocial impact align with previous studies reporting improved psychosocial outcomes over time (Jelen et al., 2023; Lemos et al., 2020; Su et al., 2014; Li et al., 2017). Our results suggest that over time, the awareness of an untreated UIA has little impact on the psychosocial outcomes. However, it's important to note that our results do not contradict previous studies reporting negative impacts in the initial period after diagnosis.

All patients in our sample had neurosurgery outpatient clinic visits at the time of study entry, with an acknowledged emphasis on minimizing distress. Algra et al. reported that counselling for UIA patients at outpatient clinics positively influences HRQOL (Algra et al., 2022). Jelen et al. demonstrated that the manner in which information about UIA diagnosis is conveyed influences patients' feelings toward the diagnosis, with negative reports following diagnosis communication over the phone, at the GP, or by mail (Jelen et al., 2023). Hence, a positive impact of diagnosis communication at the outpatient clinic may potentially be reflected in our study's results. Our results may not reflect the impact of chance findings outside established contexts.

Exempt increased use of radiology, we found no substantial impact on use of healthcare services. To our knowledge, no other studies have investigated the associations between untreated UIAs and use of health services and work absenteeism, providing no basis for comparison. While there was a tendency toward more sick leave among cases compared to controls, the differences did not reach statistical significance, potentially due to a small sample size. Further studies with larger sample sizes and representative age groups are warranted.

## 6 Conclusions

Our study suggests that diagnoses of small UIAs that are left untreated have minimal impact on long-term psychosocial outcomes and the use of healthcare services. However, due to potential selection bias and a relatively small sample size with mainly older participants, our findings may not be fully generalisable to broader populations outside the health survey context.

## Data availability statement

No additional data available. Individual patient level data are protected by law. Data can be accessed by application to the respective registries.

## Contributors

TI and JI had the original idea for this study. All authors participated in the study's design. IMR obtained access to registry and survey data, collected follow-up data, conducted statistical analyses, and drafted the manuscript. All authors contributed to interpreting the results and writing the final manuscript.

## Ethics

Ethical approval was obtained from Regional Committees for Medical and Health Research Ethics (REC North, ref. Number 126174) and written consent was obtained from all participants. Invited participants consented to participation in Tromsø7, including further research and linkage to health registries (REC North, ref. Number 2014/940).

## Funding

This work received support from the Northern Norway Regional Health Authority, grant number HNF1542-20. The Northern Norway Regional Health Authority had no role in the study design, data collection, analysis, interpretation, or writing of the manuscript.

## Patient involvement

The Norwegian Heart and Lung Patient Organization was involved in planning of the study, interpretation of results, and manuscript feedback.

## Declaration of generative AI and AI-assisted technologies in the writing process

During the preparation of this work the authors used ChatGPT in order to improve language quality. After using this tool/service, the authors reviewed and edited the content as needed and takes full responsibility for the content of the publication.

## Declaration of competing interest

The authors declare that they have no known competing financial interests or personal relationships that could have appeared to influence the work reported in this paper.

## References

[bib1] Algra A.M., Lindgren A., Vergouwen M.D.I. (2019). Procedural clinical complications, case-fatality risks, and risk factors in endovascular and neurosurgical treatment of unruptured intracranial aneurysms: a systematic review and meta-analysis. JAMA Neurol..

[bib2] Algra A.M., Greving J.P., Wermer M.J. (2022). Quality of life outcomes over time in patients with unruptured intracranial aneurysms with and without preventive occlusion: a prospective cohort study. Neurology.

[bib3] Buijs J.E., Greebe P., Rinkel G.J. (2011). Quality of life, anxiety, and depression in patients with an unruptured intracranial aneurysm with or without aneurysm occlusion. Neurosurgery.

[bib4] Chen P., Lin K.C., Liing R.J. (2016). Validity, responsiveness, and minimal clinically important difference of EQ-5D-5L in stroke patients undergoing rehabilitation. Qual. Life Res..

[bib5] Devlin N.J., Shah K.K., Feng Y. (2018). Valuing health-related quality of life: an EQ-5D-5L value set for England. Health Econ..

[bib6] Eggen A.E., Mathiesen E.B., Wilsgaard T. (2013). The sixth survey of the Tromsø Study (Tromsø 6) in 2007–08: collaborative research in the interface between clinical medicine and epidemiology: study objectives, design, data collection procedures, and attendance in a multipurpose population-based health survey. Scand. J. Publ. Health.

[bib7] Gabriel R.A., Kim H., Sidney S. (2010). Ten-year detection rate of brain arteriovenous malformations in a large, multiethnic, defined population. Stroke.

[bib8] Grepperud S. (2018). Private behandlingsforsikringer–status og mulige konsekvenser på effektivitet og fordeling [Private Health Insurance - status and Potential Implications on Efficiency and Distribution]. Health Econ. Res. Netw..

[bib9] Greving J.P., Wermer M.J., Brown R.D. (2014). Development of the PHASES score for prediction of risk of rupture of intracranial aneurysms: a pooled analysis of six prospective cohort studies. Lancet Neurol..

[bib10] (2023). Helfo. Endringer i regelverk og takster fra 1. januar 2023 [Changes in regulations and tariffs from 1 January 2023]. Tønsberg: Helfo.

[bib11] Hopstock L.A., Grimsgaard S., Johansen H. (2022). The seventh survey of the Tromso Study (Tromso7) 2015-2016: study design, data collection, attendance, and prevalence of risk factors and disease in a multipurpose population-based health survey. Scand. J. Publ. Health.

[bib12] Ignacio K.H.D., Pascual J.S.G., Factor S.J.V., Khu K.J.O. (2022). A meta-analysis on the prevalence of anxiety and depression in patients with unruptured intracranial aneurysms: exposing critical treatment gaps. Neurosurg. Rev..

[bib13] Jacobsen B.K., Eggen A.E., Mathiesen E.B. (2012). Cohort profile: the tromso study. Int. J. Epidemiol..

[bib14] Jelen M.B., Clarke R.E., Jones B. (2023). Psychological and functional impact of a small unruptured intracranial aneurysm diagnosis: a mixed‐methods evaluation of the patient journey. Stroke: Vascular and Interventional Neurology.

[bib15] Johnsen L.H., Herder M., Vangberg T. (2022). Prevalence of unruptured intracranial aneurysms: impact of different definitions - the Tromso Study. J. Neurol. Neurosurg. Psychiatry.

[bib16] Lemos M., Roman-Calderon J.P., Calle G. (2020). Personality and anxiety are related to health-related quality of life in unruptured intracranial aneurysm patients selected for non-intervention: a cross sectional study. PLoS One.

[bib17] Li Y., Dai W., Zhang J. (2017). Anxiety, depression and quality of life in patients with a treated or untreated unruptured intracranial aneurysm. J. Clin. Neurosci..

[bib18] Macdonald R.L., Schweizer T.A. (2017). Spontaneous subarachnoid haemorrhage. Lancet.

[bib19] Malhotra A., Wu X., Forman H.P. (2017). Growth and rupture risk of small unruptured intracranial aneurysms: a systematic review. Ann. Intern. Med..

[bib20] McCaffery K.J., Barratt A.L. (2004). Assessing psychosocial/quality of life outcomes in screening: how do we do it better?. J. Epidemiol. Community Health.

[bib21] Morita A. (2019). Value of brain dock (brain screening) system in Japan. World Neurosurg.

[bib22] Rabin R., de Charro F. (2001). EQ-5D: a measure of health status from the EuroQol Group. Ann. Med..

[bib23] Randall D.J., Zhang Y., Li H. (2022). Establishing the minimal clinically important difference and substantial clinical benefit for the pain visual analog scale in a postoperative hand surgery population. J Hand Surg Am.

[bib24] Scan.com. Available from: https://uk.scan.com/.

[bib25] Smith-Bindman R., Kwan M.L., Marlow E.C. (2019). Trends in use of medical imaging in US health care systems and in ontario, Canada, 2000-2016. JAMA.

[bib26] StataCorp. Stata Statistical Software: Release 17,. College Station, TX: StataCorp LLC; 2019.

[bib27] Strand B.H., Dalgard O.S., Tambs K., Rognerud M. (2003). Measuring the mental health status of the Norwegian population: a comparison of the instruments SCL-25, SCL-10, SCL-5 and MHI-5 (SF-36). Nord. J. Psychiatr..

[bib28] Su S.H., Xu W., Hai J. (2014). Cognitive function, depression, anxiety and quality of life in Chinese patients with untreated unruptured intracranial aneurysms. J. Clin. Neurosci..

[bib29] The Norwegian Directorate of eHealth (2018).

[bib30] The Norwegian Medical Association (2022).

[bib31] Thompson B.G., Brown R.D., Amin-Hanjani S. (2015). Guidelines for the management of patients with unruptured intracranial aneurysms: a guideline for healthcare professionals from the American heart association/American stroke association. Stroke.

[bib32] van der Schaaf I.C., Brilstra E.H., Rinkel G.J. (2002). Quality of life, anxiety, and depression in patients with an untreated intracranial aneurysm or arteriovenous malformation. Stroke.

[bib33] Veddegjaerde K.E., Sivertsen B., Wilhelmsen I., Skogen J.C. (2014). Confirmatory factor analysis and item response theory analysis of the Whiteley Index. Results from a large population based study in Norway. The Hordaland Health Study (HUSK). J. Psychosom. Res..

[bib34] Vlak M.H., Algra A., Brandenburg R., Rinkel G.J. (2011). Prevalence of unruptured intracranial aneurysms, with emphasis on sex, age, comorbidity, country, and time period: a systematic review and meta-analysis. Lancet Neurol..

[bib35] von Elm E., Altman D.G., Egger M. (2008). The Strengthening the Reporting of Observational Studies in Epidemiology (STROBE) statement: guidelines for reporting observational studies. J. Clin. Epidemiol..

[bib36] Wermer M.J., van der Schaaf I.C., Algra A., Rinkel G.J. (2007). Risk of rupture of unruptured intracranial aneurysms in relation to patient and aneurysm characteristics: an updated meta-analysis. Stroke.

[bib37] World Health Organization. European Health Information Gateway. Available from:: https://gateway.euro.who.int/en/.

