# Supplementary material for: Are diagnoses of unruptured intracranial aneurysms associated with quality of life, psychological distress, health anxiety, or use of healthcare services in untreated individuals? A longitudinal, nested case-control study
Source: Brain Spine. 2024 Aug 13;4:102915. doi: 10.1016/j.bas.2024.102915 (PMC11386048; doi:10.1016/j.bas.2024.102915)
Supplement: Multimedia component 1 [file mmc1.docx]

*Supplemental file 1. Use of medication following diagnosis of unruptured intracranial aneurysm.*

|  | Cases | | Control1 | | P value^a^ | Control2 | | P value^a^ |
| --- | --- | --- | --- | --- | --- | --- | --- | --- |
|  | Baseline | Follow-up | Baseline | Follow-up |  | Baseline | Follow-up |  |
| Blood pressure-lowering drugs, % (n/total) | 31.1 (23/74) | 40.5 (30/74) | 26.5 (39/147) | 35.4 (52/147) | 0.73 | 24.8 (33/133) | 37.6 (50/133) | 0.73 |
| Cholesterol lowering-drugs, % (n/total) | 27.4 (20/73) | 34.2 (25/73) | 25.0 (37/148) | 29.1 (43/148) | 0.47 | 18.8 (25/133) | 27.1 (36/133) | 0.73 |
| Diuretics, % (n/total) | 1.4 (1/71) | 2.8 (2/71) | 2.1 (3/145) | 4.1 (6/145) | 0.73 | 7.8 (10/128) | 9.4 (12/128) | 0.45 |
| Drugs for heart disease, % (n/total) | 17.1 (12/70) | 20.0 (14/70) | 15.8 (23/146) | 19.9 (29/146) | 0.86 | 11.5 (15/130) | 23.1 (30/130) | 0.18 |
| Insulin, % (n/total) | 4.3 (3/69) | 2.9 (2/69) | 2.8 (4/143) | 3.5 (5/143) |  | 0  (0/126) | 0  (0/126) |  |
| Tablets for diabetes, % (n/total) | (5/68) | (5/68) | (5/143) | (5/143) | 0.78 | (6/129) | (8/129) | 0.56 |
| Drugs for hypothyroidism (Levaxin or thyroxine), % (n/total) | 11.8 (8/68) | 11.8 (8/68) | 5.6 (8/144) | 6.9 (10/144) | 0.66 | 13.3 (17/128) | 14.1 (18/128) | 0.64 |
| Painkillers during the last 4 weeks, % (n/total) | 43.1 (31/72) | 52.8 (38/72) | 50.7 (75/148) | 48.6 (72/148) | 0.25 | 54.5 (73/134) | 50.7 (68/134) | 0.28 |
| Sleeping pills, tranquilizers, or antidepressants during the last 4 weeks, % (n/total) | 14.1 (10/71) | 21.1 (15/71) | 11.5 (16/139) | 16.5 (23/139) | 0.53 | 11.7 (15/128) | 14.8 (19/128) | 0.31 |

^a^ Separate logistic regression analyses of differences between cases and the two control groups at follow-up, adjusted for corresponding baseline values.
